# Vectorborne Infections, Mali

**DOI:** 10.3201/eid2202.150688

**Published:** 2016-02

**Authors:** David Safronetz, Moussa Sacko, Nafomon Sogoba, Kyle Rosenke, Cynthia Martellaro, Sékou Traoré, Issa Cissé, Ousmane Maiga, Matt Boisen, Diana Nelson, Darin Oottamasathien, Molly Millett, Robert F. Garry, Luis M. Branco, Seydou Doumbia, Heinz Feldmann, Mamadou S. Traoré

**Affiliations:** National Institutes of Health, Hamilton, Montana, USA (D. Safronetz, K. Rosenke, C. Martellaro, H. Feldmann);; Institut National de Recherche en Sante Publique, Bamako, Mali (M. Sacko, S. Traoré, I. Cissé, M.S. Traoré);; University of Sciences, Techniques and Technologies of Bamako, Bamako (N. Sogoba, O. Maiga, S. Doumbia);; Corgenix Medical Corporation, Inc., Broomfield, Colorado, USA (M. Boisen, D. Nelson, D. Oottamasathien, M. Millett);; Tulane School of Medicine, New Orleans, Louisiana, USA (R.F. Garry);; Zalgen Labs, LLC, Germantown, Maryland, USA (L.M. Branco)

**Keywords:** zoonotic diseases, Leptospira, chikungunya virus, dengue virus, Crimean-Congo hemorrhagic fever virus, hantavirus, Lassa virus, West Nile virus, Ebola virus, arboviruses, rodentborne viruses, viruses, vector-borne infections, flavivirus, bunyavirus, togavirus

**To the Editor:** As in many West Africa nations, vectorborne diseases represent a substantial health burden in Mali; however, beyond malaria, the incidence and etiology of many of these diseases is poorly understood. Of the estimated 14.1 million persons living in sub-Saharan Mali, ≈70% live in remote rural settings with an ecologic landscape that puts inhabitants at an increased risk for contact with rodent and arthropodborne diseases. We retrospectively analyzed serum samples for evidence of recent (IgM+) and previous (IgG+) infection with chikungunya (CHIKV), dengue (DENV), West Nile (WNV), Lassa (LASV), Crimean-Congo hemorrhagic fever (CCHFV), and Ebola (EBOV) virus, as well as Old World hantaviruses (OW-HANV) and *Leptospira* spp., which is regularly misdiagnosed as an acute viral infection.

We tested 376 deidentified serum samples collected from acutely ill patients who had a history of fever and hemorrhagic, diarrheal, or icteric syndromes (Technical Appendix Figure). Research on samples from humans was conducted in accordance with the policies and regulations of the National Institutes of Health and adhered to the principles of the Belmont Report (1979) (http://www.hhs.gov/ohrp/humansubjects/guidance/belmont.html). This research was conducted under an institutional review board–approved document.

Samples had previously tested negative for acute *Plasmodium falciparum* malaria and yellow fever virus infections. Commercially available IgM capture and conventional IgG ELISAs were used for serologic testing for CHIKV (GenWay Biotech, San Diego, CA, USA); DENV (all four serotypes) and WNV (both from Focus Diagnostics, Cypress, CA, USA); OW-HANVs (Euroimmun, Luebeck, Germany); and *Leptospira* spp. (Abnova, Taipei City, Taiwan). Conventional IgM/IgG ELISAs were used for LASV (Corgenix, Broomfield, CO, USA) and CCHFV (Vector-Best, Novosibirsk, Russia), and reagents for the EBOV IgM/IgG ELISA (infected/uninfected cell lysates) were prepared at the Rocky Mountain Laboratories (Hamilton, MT, USA) and validated with serum from experimentally infected monkeys. With the exception of the CHIKV, *Leptospira* spp., and in-house EBOV assays, the tests conducted in this study are under preclinical development for human diagnostic assays.

Samples were tested at a 1:100 dilution according to manufacturer specifications (CHIKV, CCHFV, WNV, DENV, OW-HANVs, LASV, and *Leptospira* spp.) or in-house quality-control assessments (EBOV), in a blinded fashion. Serologic reactivity was assessed according to manufacturer recommendations. For the EBOV ELISA, samples were deemed positive if optical density at 405 nm was >3 SD above that of the average of known negative samples.

Serologic evidence suggestive of acute infection (IgM+) with 1 of the pathogens tested for was observed for 39.9% of samples (Table). At 14.4%, *Leptospira* spp. was the most prevalent probable etiologic agent of acute disease identified. Of mosquitoborne viruses tested, DENV had the highest prevalence at 7.7%, followed by CHIKV (5.3%) and WNV (0.27%). Of rodentborne pathogens, OW-HANVs had a seroprevalence of 7.2%, whereas LASV was considerably lower (0.27%). CCHFV IgM was documented in 4.8% of samples. Overall, little annual variation in the IgM seroprevalence was noted, except for *Leptospira* spp., for which 2 obvious peaks in seroprevalence were observed (Table).

**Table:** IgM and IgG seroprevalence rates of selected vectorborne pathogens in samples submitted from suspected yellow fever cases in Mali, 2009–2013*

Pathogen	IgM/IgG positivity	No. (%) samples					
		2009, n = 77	2010, n = 107	2011, n = 71	2012, n = 34	2013, n = 87	Total, N = 376
Chikungunya virus	IgM+	4 (5.2)	4 (3.7)	5 (7.0)	2 (5.9)	5 (5.7)	20 (5.3)
IgG+	5 (6.5)	9 (8.4)	2 (2.8)	2 (5.9)	7 (8.0)	25 (6.6)
IgG+/IgM+	0	1	0	0	0	1

West Nile virus	IgM+	0	0	0	0	1 (1.1)	1 (0.27)
IgG+	33 (42.9)	46 (43.0)	28 (39.4)	13 (38.2)	27 (31.0)	147 (39.1)
IgG+/IgM+	0	0	0	0	0	0

Dengue virus	IgM+	6 (7.8)	11 (10.3)	4 (5.6)	4 (11.8)	4 (4.6)	29 (7.7)
IgG+	32 (41.6)	46 (43.0)	31 (43.7)	13 (38.2)	28 (32.2)	150 (40.0)
IgG+/IgM+	3	6	1	1	2	13

*Leptospira* spp.	IgM+	7 (9.1)	23 (21.5)	1 (1.4)	3 (8.8)	20 (23.0)	54 (14.4)
IgG+	12 (15.6)	19 (17.8)	15 (21.1)	8 (23.5)	20 (23.0)	74 (19.7)
IgG/IgM+	1	2	1	0	3	7

OW-HANV	IgM+	5 (6.5)	6 (6.5)	5 (7.0)	2 (6.7)	7 (8.0)	27 (7.2)
IgG+	2 (2.6)	8 (7.5)	6 (8.5)	2 (6.7)	3 (3.4)	21 (5.6)
IgG+/IgM+	0	0	0	0	0	0

Lassa virus	IgM+	0	0	0	0	1 (1.1)	1 (0.27)
IgG+	0	0	0	0	0	0
IgG+/IgM+	0	0	0	0	0	0

CCHFV	IgM+	5 (6.5)	5 (4.7)	2 (2.8)	0	6 (6.9)	18 (4.8)
IgG+	2 (2.6)	3 (2.8)	2 (2.8)	1 (2.9)	3 (3.4)	11 (2.9)
IgG+/IgM+	0	0	0	0	0	0

Ebola virus	IgM+	0	0	0	0	0	0
IgG+	0	0	0	0	0	0
IgG+/IgM+	0	0	0	0	0	0

*OW-HANV, Old World hantavirus; CCHFV, Crimean-Congo hemorrhagic fever virus.

Most IgM+ samples demonstrated serologic reactivity in only 1 assay. The exception was 2 samples that were IgM+ for hantaviruses and *Leptospira* spp., an acute dual infection that might be underrecognized (*1*). With the exception of DENV, few samples were both IgM+ and IgG+, suggesting the results were not attributable to IgM persistence. The DENV IgM+/IgG+ results might represent IgM persistence. However, because the ELISA detected all 4 serotypes, it is plausible that some results represent recent infection with DENV in the presence of IgG reactive with a different serotype. 

The relatively high IgG seroprevalence for most of the pathogens tested supports the findings of the IgM assays and further suggest the circulation of and potential for human exposure to these agents in Mali (Table). Geographically, serologic evidence of infections with *Leptospira* spp., DENV, WNV, OW-HANVs, and CHIKV was observed throughout Mali (online Technical Appendix). No samples were reactive with EBOV, and the low incidence of LASV infection is not surprising because the samples analyzed here were collected outside of the 1 documented LASV-endemic region in Mali (*2*).

We used commercially available diagnostic platforms, primarily IgM capture and conventional IgG ELISAs, many of which are validated for human diagnostics. Ideally, diagnostics for zoonotic diseases would not rely on IgM/IgG serologic analysis because of caveats including IgM persistence and cross-reactivity between closely related pathogens (*3*,*4*). In the industrialized world, as well as in several countries throughout Africa, molecular approaches are often used to genetically identify pathogens, or follow-up convalescent-phase serum samples are collected to determine seroconversion or increased antibody titers or to conduct plaque reduction neutralization assays. Unfortunately, because of the nature of the samples available, including time of collection, storage history, and remaining volume, many of these tests were not feasible for our study.

Despite these limitations, these serologic findings indicate that flaviviruses, bunyaviruses, and togaviruses, as well as *Leptospira* spp., are contributing to human illness in Mali. These results add to those recently documented in studies conducted in Sierra Leone, implying that several of these zoonotic pathogens are widely distributed yet underreported throughout West Africa (*5*,*6*).

**Technical Appendix.** Distribution of serum samples tested, by region and year. IgM and IgG seroprevalence rates of selected vectorborne pathogens, by region of sample collection.
