# Supplementary material for: Vectorborne Infections, Mali
Source: Emerg Infect Dis. 2016 Feb;22(2):340–2. doi: 10.3201/eid2202.150688 (PMC4734548; doi:10.3201/eid2202.150688)
Supplement: Supplementary file 1 — Technical Appendix. Distribution of serum samples tested, by region and year. IgM and IgG seroprevalence rates of selected vectorborne pathogens, by region of sample collection. [file 15-0688-Techapp-s1.pdf]

Article DOI: http://dx.doi.org/10.3201/eid2202.150688

# Vectorborne Infections, Mali

## Technical Appendix

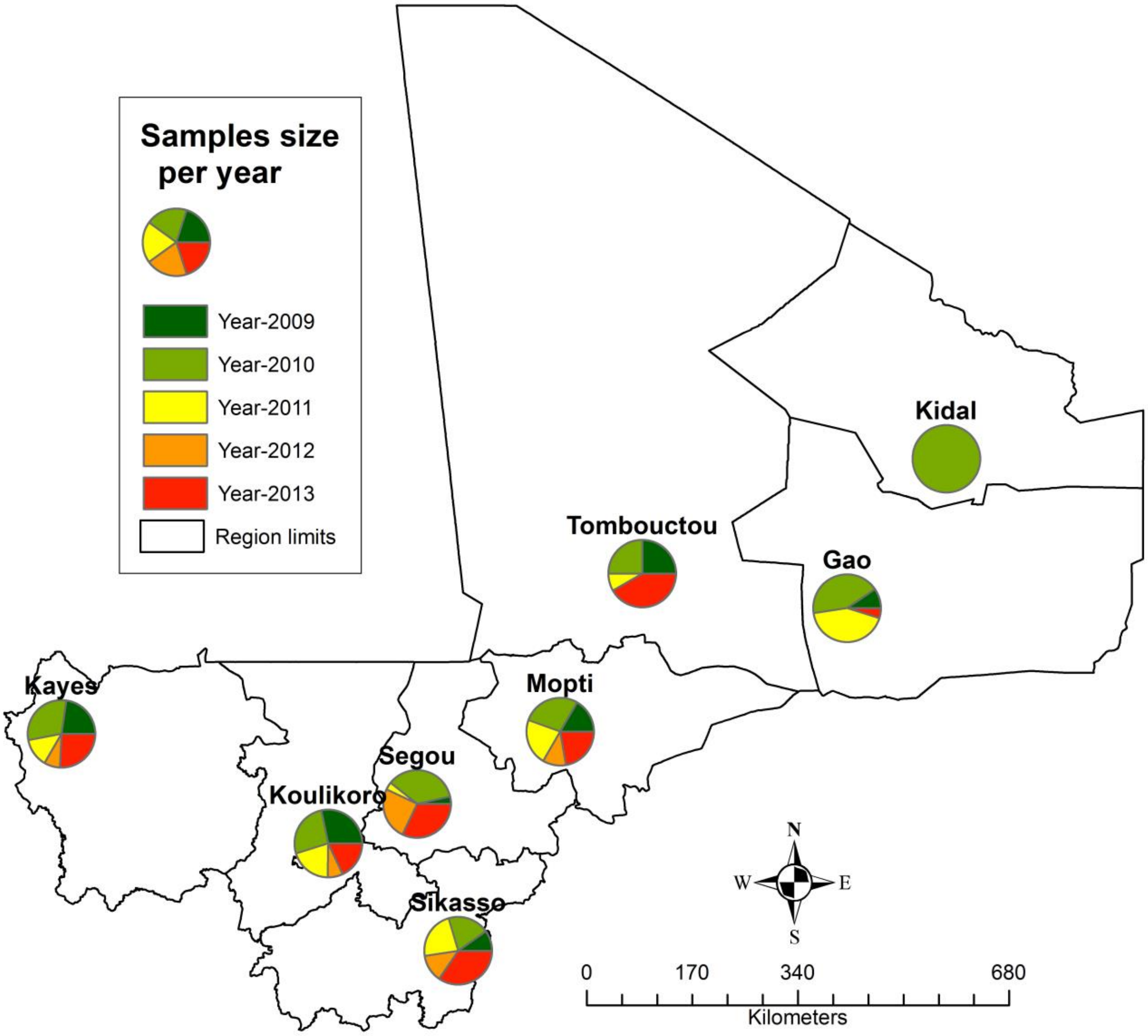


**Technical Appendix Figure.** Distribution of samples tested (N = 376), by region and year, Mali. Samples from Kayes (n = 66), Koulikoro (n = 166), Sikasso (n = 61), Segou (n = 28), Mopti (n = 18), Timbuktu (n = 12), Gao (n = 21), and Kidal (n = 4) were tested for serologic evidence of infection with 8 zoonotic pathogens.

**Technical Appendix Table.** IgM and IgG seroprevalence rates of selected vectorborne pathogens, by region of sample collection, Mali*

| | | No. (%) sample | | | | | | | |
|---|---|---|---|---|---|---|---|---|---|
| Pathogen | Assay | Kayes, n = 66 | Koulikoro, n = 166 | Sikasso, n = 61 | Segou, n = 28 | Mopti, n = 18 | Timbuktu, n = 12 | Gao, n = 21 | Kidal, n = 4 |
| Chikungunya virus | IgM | 4 (6.1) | 9 (6.0) | 2 (3.3) | 2 (7.1) | 1 (5.6) | 1 (8.3) | 1 (4.8) | 0 |
| | IgG | 10 (15.2) | 11 (6.6) | 2 (3.3) | 2 (7.1) | 0 | 0 | 0 | 0 |
| West Nile virus | IgM | 0 | 0 | 0 | 0 | 1 (5.6) | 0 | 0 | 0 |
| | IgG | 40 (60.6) | 51 (30.7) | 8 (13.1) | 12 (43) | 11 (61.1) | 8 (66.7) | 17 (81) | 0 |
| Dengue virus | IgM | 7 (10.1) | 11 (6.6) | 6 (9.8) | 1 (3.6) | 1 (5.6) | 1 (8.3) | 1 (4.8) | 1 (25) |
| | IgG | 41 (62.1) | 56 (33.7) | 10 (16.4) | 12 (42.9) | 10 (55.6) | 3 (25) | 16 (72.7) | 2 (50) |
| *Leptospira* spp. | IgM | 10 (15.2) | 23 (13.9) | 9 (14.8) | 7 (25.0) | 1 (5.6) | 3 (25.0) | 1 (4.8) | 0 |
| | IgG | 12 (18.2) | 40 (24.1) | 7 (11.5) | 6 (21.4) | 3 (16.7) | 2 (16.7) | 4 (19) | 0 |
| OW-HANV | IgM | 5 (7.6) | 12 (7.2) | 5 (8.2) | 1 (3.6) | 0 | 1 (8.3) | 3 (14.3) | 0 |
| | IgG | 1 (<0.01) | 11 (6.6) | 4 (6.6) | 4 (14.3) | 0 | 0 | 0 | 1 (25) |
| Lassa virus | IgM | 0 | 0 | 0 | 0 | 0 | 1 (8.3) | 0 | 0 |
| | IgG | 0 | 0 | 0 | 0 | 0 | 0 | 0 | 0 |
| CCHFV | IgM | 4 (6.1) | 9 (5.4) | 1 (1.6) | 3 (10.7) | 0 | 1 (8.3) | 0 | 0 |
| | IgG | 5 (7.6) | 1 (0.6) | 1 (1.6) | 1 (3.6) | 0 | 3 (25) | 0 | 0 |
| Ebola virus | IgM | 0 | 0 | 0 | 0 | 0 | 0 | 0 | 0 |
| | IgG | 0 | 0 | 0 | 0 | 0 | 0 | 0 | 0 |

*OW-HANV, Old World hantavirus; CCHFV, Crimean-Congo hemorrhagic fever virus.
